# Influence of a probiotic soy product on fecal microbiota and its association with cardiovascular risk factors in an animal model

**DOI:** 10.1186/1476-511X-10-126

**Published:** 2011-07-29

**Authors:** Daniela CU Cavallini, Juliana Y Suzuki, Dulcinéia SP Abdalla, Regina C Vendramini, Nadiége D Pauly-Silveira, Mariana N Roselino, Roseli A Pinto, Elizeu A Rossi

**Affiliations:** 1 Department of Food & Nutrition, School of Pharmaceutical Sciences, Sao Paulo State University, Araraquara, SP, Brazil; 2 Department of Clinical and Toxicological Analyses, School of Pharmaceutical Sciences, University of Sao Paulo, Sao Paulo, Brazil; 3 Department of Clinical Analysis, School of Pharmaceutical Sciences, Sao Paulo State University, Araraquara, SP, Brazil

**Keywords:** probiotics, *Enterococcus faecium *CRL 183, fecal microbiota, lipid parameters

## Abstract

**Background:**

Previous work showed that daily ingestion of an aqueous soy extract fermented with *Enterococcus faecium *CRL 183 and *Lactobacillus helveticus *416, supplemented or not with isoflavones, reduced the total cholesterol and non-HDL-cholesterol levels, increased the high-density lipoprotein (HDL) concentration and inhibited the raising of autoantibody against oxidized low-density lipoprotein (ox-LDL Ab) and the development of atherosclerotic lesions.

**Objective:**

The aim of this study was to characterize the fecal microbiota in order to investigate the possible correlation between fecal microbiota, serum lipid parameters and atherosclerotic lesion development in rabbits with induced hypercholesterolemia, that ingested the aqueous soy extract fermented with *Enterococcus faecium *CRL 183 and *Lactobacillus helveticus *416.

**Methods:**

The rabbits were randomly allocated to five experimental groups (n = 6): control (C), hypercholesterolemic (H), hypercholesterolemic plus unfermented soy product (HUF), hypercholesterolemic plus fermented soy product (HF) and hypercholesterolemic plus isoflavone-supplemented fermented soy product (HIF). Lipid parameters and microbiota composition were analyzed on days 0 and 60 of the treatment and the atherosclerotic lesions were quantified at the end of the experiment. The fecal microbiota was characterized by enumerating the *Lactobacillus *spp., *Bifidobacterium *spp., *Enterococcus *spp., Enterobacteria and *Clostridium *spp. populations.

**Results:**

After 60 days of the experiment, intake of the probiotic soy product was correlated with significant increases (P < 0.05) on *Lactobacillus *spp., *Bifidobacterium *spp. and *Enterococcus *spp. and a decrease in the Enterobacteria population. A strong correlation was observed between microbiota composition and lipid profile. Populations of *Enterococcus *spp., *Lactobacillus *spp. and *Bifidobacterium *spp. were negatively correlated with total cholesterol, non-HDL-cholesterol, autoantibodies against oxidized LDL (oxLDL Ab) and lesion size. HDL-C levels were positively correlated with *Lactobacillus *spp., *Bifidobacterium *spp., and *Enterococcus *spp. populations.

**Conclusion:**

In conclusion, daily ingestion of the probiotic soy product, supplemented or not with isoflavones, may contribute to a beneficial balance of the fecal microbiota and this modulation is associated with an improved cholesterol profile and inhibition of atherosclerotic lesion development.

## Background

The human intestinal microbiota plays an important role in maintaining human health, preventing colonization by pathogenic microorganisms, breaking down dietary compounds, producing nutrients and keeping the intestinal mucosa in a healthy state. Other important functions have begun to be unveiled over the past few years, suggesting that the effects of the gut microbiota may be more profound, influencing complex processes such as glucose and lipid metabolism, predisposition to obesity and disorders mediated by the immune system, including inflammatory bowel disease, autoimmune conditions and allergic reactions [1,2].

Cardiovascular diseases (CVD) are the main cause of death around the world and dyslipidemia is a key factor in susceptibility to coronary heart disease (CHD) and other atherosclerotic diseases [3].

Recent research has afforded strong evidence suggesting that the gut microbiota could influence host cholesterol metabolism [4]. The mechanisms involved in this effect include modifications of bile acids that affect enterohepatic circulation, de novo synthesis of bile acids and cholesterol absorption and inhibition of lipoprotein lipase [5,6].

In previous studies, we have shown that daily ingestion of an aqueous soy extract fermented with *Enterococcus faecium *CRL 183 and *Lactobacillus helveticus *416, supplemented or not with isoflavones, could improve the lipid profile in animal and human tests [7-10]. Using New Zealand rabbits, Cavallini et al. (2009) [11] demonstrated that the same probiotic product, with or without isoflavones, leads to a significant reduction of serum total cholesterol and non-high density lipoprotein-cholesterol (non-HDL-C), increases the high-density lipoprotein (HDL) level, inhibition of the raise of autoantibody against oxidized low-density lipoprotein (ox-LDL Ab) and slows the development of atherosclerotic lesions. In this study, we intend to characterize the fecal microbiota of rabbits that ingest the aqueous soy extract fermented with *Enterococcus faecium *CRL 183 and *Lactobacillus helveticus *416, in order to investigate the possible correlation of specific modification of fecal microbiota, serum lipid parameters and atherosclerosis development.

## Material and Methods

### Animals and experimental protocol

Male New Zealand white rabbits (n = 30, 8-9 weeks old, weighing 2.5-3.0 Kg; obtained from Central Animal Facility of Sao Paulo State University, Botucatu, SP, Brazil) were housed in individual cages in temperature-controlled rooms (22°C ± 2°C), with a light-dark cycle of 12:12 h.

Rabbits were fed a chow diet (Purina, SP, Brazil) for 1 week to acclimatize the animals and then randomly allocated to five experimental groups (n = 6): control (C), hypercholesterolemic (H), hypercholesterolemic plus unfermented soy product (HUF), hypercholesterolemic plus fermented soy product (HF) and hypercholesterolemic plus isoflavone-supplemented fermented soy product (HIF).

The control group (C) was fed only on commercial rabbit food (Nutri Rabbit Special Chow Purina; nutritional make-up per 100 g: 23 g protein, 4 g fats, 49 g carbohydrates, 5 g fiber and 10 g minerals). The other groups (H, HUF, HF and HIF) were fed on the same rabbit diet, to which cholesterol (Sigma C 8503) had been added to induce hypercholesterolemia. The level of cholesterol added to the diet was adjusted during the experiment (1.0% down to 0.7% after 30 days), to maintain the animal healthy and the feed was prepared as previously described (Cavallini et al., 2009). The rabbits received restrict amounts (125 g/d) of each diet and had free access to water during the experimental period.

The fermented soy product was processed at the Development and Production Unit for Soybean Derivatives (UNISOJA, Food and Nutrition Department of the School of Pharmaceutical Sciences, UNESP at Araraquara - SP, Brazil), using the method described by Rossi et al (1989). The bacterial inoculum consisted of 3% (v/v) of a 1:1 mixture of *Enterococcus faecium *CRL 183 (probiotic microorganism, from *Centro de Referencia para Lactobacilos *- CERELA - Argentina) and *Lactobacillus helveticus *416 (fermentation adjuvant, from Institute of Food Technology - ITAL - Campinas, Brazil). Isoflavone-suplemented fermented soy product was prepared by adding Isoflavin^® ^(Galena, Brazil), before the fermentation, at 75 mg (total isoflavone) per 100 g. Fermented soy product, supplemented or not with isoflavones, exhibited cell viable counts between 10^9 ^- 10^10 ^CFU/mL. Unfermented soy product was prepared by chemical acidification of soy product basic mixture (without bacterial culture or isoflavone), to reach the same pH as the fermented soy product (4.4 - 4.6), by adding food-grade lactic acid (Synth, Sao Paulo, SP, Brazil). The chemical composition of the products is presented in Table 1.

**Table 1:** Composition of unfermented soy product, fermented soy product and isoflavone-supplemented fermented soy product

Composition	Unfermented Soy Product	Fermented Soy Product	Isoflavone-Supplemented Soy Product
Protein (g/100 g)	3.85 ± 0.04	3.90 ± 0.00	3.85 ± 0.00
Fat (g/100 g)	2.32 ± 0.02	2.30 ± 0.08	2.26 ± 0.09
Carbohydrate (g/100 g)	9.93 ± 0.16	9.70 ± 0.15	10.06 ± 0.11
Ash (g/100 g)	0.90 ± 0.00	0.90 ± 0.07	0.90 ± 0.00
Moisture(g/100 g)	83.00 ± 0.16	83.20 ± 0.00	82.93 ± 0.17
Total isoflavone (mg/100 g)	8.03 ± 0.07	8.04 ± 0.01	51.26 ± 1.12
Daidzin (mg/100 g)	2.00 ± 0.03	2.09 ± 0.04	4.68 ± 0.27
Genistin (mg/100 g)	5.76 ± 0.05	5.69 ± 0.04	6.72 ± 0.02
Daidzein (mg/100 g)	0.26 ± 0.01	0.26 ± 0.01	32.62 ± 0.94
Genistein (mg/100 g)	-	-	7.25 ± 0.22
Final pH	4.6	4.45	4.55

Values represent mean ± SEM (n = 3). Fermented soy product and isoflavone-supplemented soy product were fermented by a mixed culture of *Enterococcus faecium *CRL 183 and *Lactobacillus helveticus *416.

Groups HUF, HF and HIF received, by gavage once a day, unfermented soy product (2.8 mL/kg body weight), fermented soy product (2.8 mL/kg body weight) and isoflavone-supplemented fermented soy product (2.8 mL/kg body weight - 2.1 mg of total isoflavone/kg body weight), respectively.

The experiment was carried out for 60 days. The fecal microbiota, lipid parameters and atherosclerotic lesion development were analyzed before the start (T0) and at the end of the experimental period (T60).

The experimental design received approval (n° 03/2007) from the Research Ethics Committee of the School of Pharmaceutical Sciences, UNESP at Araraquara (SP, Brazil).

### Stool collection

Stools were collected from the cages for 24 hours at the each sampling time (days 0 and 60). Samples were collected in aseptic plastic bags, placed in anaerobic jars with gas-generating kits (Anaerobac, Probac, Brazil) and then analyzed for bacterial counts as soon as possible, as described below.

### Blood Sampling

Blood was drawn from the marginal ear vein, after a 14 to 16-hour fast. The samples were centrifuged (3500 x g for 10 min) and the serum taken for lipid profile determination. For the oxLDL Ab determination, plasma was first separated from the blood by centrifugation (3500 × g for 10 min at 4°C), then 1.0 mmol/L phenylmethylsulfonyl fluoride (Sigma Chemical), 2.0 mmol/L benzamidine (Sigma Chemical), 2.0 mg/L aprotinin (Sigma Chemical) and 20.0 mmol/L BHT (Sigma Chemical) were added to the samples of plasma.

### Analysis of Serum lipids

The serum levels of total cholesterol (TC), high-density lipoprotein (HDL-C) and triglycerides were assayed in each rabbit, with the aid of specific enzyme kits. TC was measured by the cholesterol fast color method [12]. HDL-C was estimated by first selectively precipitating lipoproteins [13] and then applying the TC method to the supernatant. Non-HDL cholesterol was calculated by subtracting HDL-C from TC and was composed of the LDL+IDL+VLDL cholesterol fractions [14,15].

### Detection of autoantibodies against oxidized LDL (oxLDL Abs)

The autoantibodies against oxLDL were assayed in plasma by ELISA, as previously described [16,17]. The data were presented as milli Arbitrary Units (mAU/L).

### Analysis of atherosclerotic lesions

After the 60 days of the experiment, the rabbits were heparinized and euthanized by an overdose of sodium phenobarbital (130 mg/kg body weight - Cristália, SP, Brazil) and the aorta was removed to analyze the macroscopic atherosclerotic lesions. The aorta was divided into the following segments: 1) aortic arch; 2) thoracic and abdominal aortas. The material was fixed overnight, at room temperature, in 10% buffered formaldehyde solution and stained with Sudan IV to reveal areas of atherosclerotic plaque [18]. The stained aorta was photographed with a digital camera (Sony) and the sudanophilic lesions were identified and quantified. The surface area of the atherosclerotic lesions was measured with an image analyzer system (Imagelab - USP - Brazil) and expressed as a percentage of the total aortic surface area covered by lesion.

### Analysis of fecal microbiota

Each fecal sample (1 g) was homogenized in a stomacher, with sterile peptone water (99 g). Subsequent tenfold serial dilutions were plated in triplicate on the following selective media to distinguish bacterial genera: *Enterococcus *spp.: KF *Streptococcus *agar (Acumedia) [19]; *Lactobacillus *spp.: MRS agar (Merck, Darmstadt, Deutschland, Germany) [20]; *Bifidobacterium *spp.: *Bifidobacterium *iodoacetate medium 25 (BIM-25) [21]; Enterobacteria: MacConkey agar (Acumedia) [22]; *Clostridium *spp.: RCA agar (Difco, Le Point de Claix, France) [23]. Plates for *Enterococcus *spp. and Enterobacteria were incubated at 37°C for 48 h. Plates for *Lactobacillus *spp., *Clostridium *spp. and *Bifidobacterium *spp were incubated anaerobically, in anaerobic jars with gas-generating kits, at 37°C for 48 h, 48 h and 72 h, respectively.

### Statistical analysis

Results are presented as means ± standard deviations. One-way ANOVA was used to determine a significant difference between groups (P < 0.05). Tukey's test was used to perform multiple comparisons between means. The difference between study periods was verified by Student's t-test. Correlations between cardiovascular risk factors and bacterial populations were assessed by Pearson's correlation test.

## Results

### Lipid parameters and atherosclerotic lesion

The effects of treatments on lipid parameters are shown in Table 2.

**Table 2:** Serum lipids and oxLDL Abs among the groups

*Serum lipids*	*Time*	C	H	HUF	HF	HIF
TC						
(mg/dL)	*T0*	49.0 ± 3.7^aA^	42.0 ± 2.8^aB^	44.8 ± 5.8^aB^	45.3 ± 5.1^aB^	44.5 ± 5.4^aB^
	T60	53.3 ± 6.2^bA^	2556.5 ± 120.3^aA^	1876.3 ± 233.6^bA^	1583.8 ± 86.3^cA^	1867.5 ± 251.7^bA^
HDL-C						
(mg/dL)	T0	31.5 ± 2.3^aA^	28.5 ± 2.5 ^aA^	31.50 ± 3.6^aA^	32.0 ± 2.6^aA^	31.8 ± 1.1^aA^
	T60	26.8 ± 2.8^aA^	16.8 ± 2.8^cB^	22.8 ± 2.8^abB^	32.0 ± 2.6^aA^	26.0 ± 2.1^aB^
n-HDL-C						
(mg/dL)	T0	17.5 ± 2.6^aB^	13.5 ± 2.9^aB^	13.3 ± 2.4^aB^	13.3 ± 3.0^aB^	12.3 ± 2.3^aB^
	T60	26.5 ± 4.4^cA^	2539.8 ± 120.2^aA^	1853.5 ± 231.8^bA^	1557.8 ± 86.6^bA^	1841.5 ± 249.8^bA^
oxLDL Abs						
(mAU/L)	T0	0.204 ± 0.104^aA^	0.339 ± 0.129^aB^	0.323 ± 0.105^aB^	0.390 ± 0.289^aA^	0.338 ± 0.166^aA^
	T60	0.316 ± 0.156^bA^	1.809 ± 0.514^aA^	0.799 ± 0.285^bA^	0.553 ± 0.296^bA^	0.530 ± 0.232^bA^

Values (in mg/dL) represent mean ± SEM (n = 6). C = control; H = hypercholesterolemic; HUF = hypercholesterolemic plus unfermented soy product, HF = hypercholesterolemic plus fermented soy product, HIF = hypercholesterolemic plus isoflavone-supplemented fermented soy product. T0 = before the start of the study, T60 = end of the experiment. Statistical comparison of treatment groups: means with identical lower-case letters in the same row do not differ significantly (P ≤ 0.05). Statistical comparison of times: means with identical upper-case letters in the same column do not differ significantly (P ≤ 0.05).

After 60 days of treatment, rabbits that received fermented soy product exhibited the greatest reduction (p < 0.05) in n-HDL-C (38.7%), relative to group H. Animal in the groups HF, HIF and HUF exhibited significantly lower TC levels than group H (38.1%, 27.0% and 26.6% respectively).

The HDL-C level was higher in animals that received the fermented soy product, supplemented or not with isoflavones (55.2%), and unfermented soy product (35.8%), than in group H. However, statistical comparison of samples at different times showed that only the HF and C groups showed no reduction in HDL-C levels after 60 days of the experiment.

The animals fed a high cholesterol diet (H) and unfermented soy product (HUF) exhibited a rapid rise in anti-oxLDL Ab (P < 0.05), while the fermented soy product (HF) and fermented soy product supplemented with isoflavones (HIF) reduced the rise in this parameter during the experiment.

**Figure 1:**
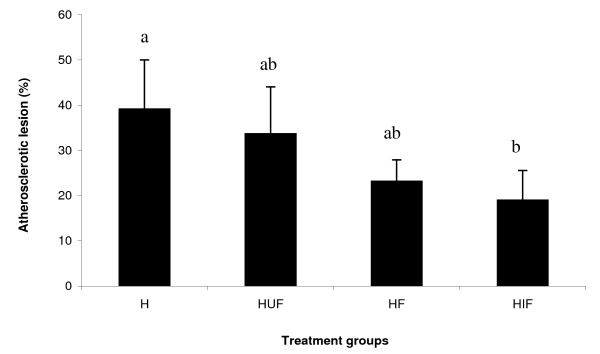
**Percentage of aortic area of whole aorta (thoracic and arch) covered with lesion**. The bar graphs represent the average (n = 6) for each group with standard errors. C = control; H = hypercholesterolemic; HUF = hypercholesterolemic plus unfermented soy product, HF = hypercholesterolemic plus fermented soy product, HIF = hypercholesterolemic plus isoflavone-suplemented soy fermented product.

Comparisons of atherosclerotic lesion area in aortic segments are shown in Figure 1. The extent of atherosclerosis on the whole aorta demonstrated that animals treated with fermented soy product supplemented with isoflavones exhibited the lower percentage of aortic area covered with lesion (19,0%), differing significantly from the group H (39,13%).

### Characterization of fecal microbiota

The fecal bacterial counts of the different treatment groups are summarized in Table 3. At the start of the protocol the microbiota compositions of the rabbits groups were different (p < 0.05), so that the comparison of times was more relevant.

**Table 3:** Fecal bacterial counts (log cfu/g) in rabbits during the experimental period

Groups	T0	T60	T0	T60
	***Enterococcus *spp.**		***Lactobacillus *spp.**	

C	5.87 ± 0.08^B^	6.90 ± 0.01^A^	6.51 ± 0.02^B^	6.99 ± 0.02^A^
H	7.26 ± 0.01^A^	6.85 ± 0.01^B^	8.07 ± 0.11^A^	7.17 ± 0.01^B^
HUF	7.23 ± 0.04^B^	7.50 ± 0.02^A^	8.92 ± 0.04^A^	8.11 ± 0.01^B^
HF	6.60 ± 0.01^B^	7.97 ± 0.02^A^	7.28 ± 0.02^B^	8.55 ± 0.05^A^
HIF	5.27 ± 0.01^B^	7.68 ± 0.03^A^	7.50 ± 0.03^B^	8.16 ± 0.02^A^

	***Bifidobacterium *spp.**		**Enterobacteria**	

C	5.46 ± 0.11^B^	6.56 ± 0.01^A^	8.81 ± 0.06^A^	7.86 ± 0.04^B^
H	8.54 ± 0.01^A^	6.91 ± 0.03^B^	8.16 ± 0.01^A^	6.71 ± 0.02^B^
HUF	9.04 ± 0.02^A^	7.93 ± 0.01^B^	8.32 ± 0.03^A^	6.96 ± 0.02^B^
HF	7.61 ± 0.01^B^	8.29 ± 0.02^A^	7.00 ± 0.05^A^	3.47 ± 0.05^B^
HIF	8.59 ± 0.03^A^	8.07 ± 0.12^B^	7.59 ± 0.23^A^	6.64 ± 0.05^B^

	***Clostridium *spp.**			

C	8.77 ± 0.05^A^	7.85 ± 0.07^B^		
H	8.69 ± 0.01^A^	7.33 ± 0.01^B^		
HUF	8.90 ± 0.02^A^	7.67 ± 0.01^B^		
HF	7.41 ± 0.05^A^	7.53 ± 0.11^A^		
HIF	8.69 ± 0.05^A^	8.04 ± 0.01^B^		

Groups: C = control, H = hypercholesterolemic, HUF = hypercholesterolemic plus unfermented soy product, HF = hypercholesterolemic plus fermented soy product, HIF = hypercholesterolemic plus isoflavone-supplemented soy fermented product. T0 = before the start of the study, T60 = end of the ingestion period. Statistical comparison of times: means with identical upper-case letters in the same row do not differ significantly (P ≤ 0.05).

After 60 days of treatment, animals that consumed only the hypercholesterolemic diet (group H) showed a significant reduction in all bacteria groups studied, including the beneficial genera *Bifidobacterium *spp. and *Lactobacillus *spp. Rabbits fed a probiotic soy product (HF) and commercial chow (C) showed a significant increase in *Bifidobacterium *spp. counts. The population of *Lactobacillus *spp. was increased in the animals that received fermented soy product, supplemented or not with isoflavones, and in the control group. Unfermented soy product was unable to increase or prevent the decrease of *Bifidobacterium *spp. and *Lactobacillus *spp. populations.

Fecal *Enterococcus *spp. population was significantly increased in the groups control (C), hypercholesterolemic plus unfermented soy product (HUF), hypercholesterolemic plus fermented soy product (HF) and hypercholesterolemic plus isoflavone-suplemented fermented soy product (HIF). All the treatment groups displayed a decrease in the Enterobacteria counts during the experiment. By the end of the study, the population of *Clostridium *spp. fell significantly in all groups, excepting in the animals of the hypercholesterolemic plus fermented soy product group (HF), compared to basal period (T0).

### Correlation of fecal microbiota and metabolic parameters

The analysis showed a strong negative correlation between serum concentrations of total cholesterol, non-HDL-C and autoantibody against oxidized LDL and area of atherosclerotic lesion, on one hand, and *Enterococcus *spp., *Lactobacillus *spp. and *Bifidobacterium *spp. counts, on the other, and a positive correlation between HDL-C and these microorganisms genera (Table 4). The Enterobacteria population was negatively correlated only with the HDL-C. Finally the levels of autoantibody against oxidized LDL and the size of atherosclerotic lesion were negatively correlated with the *Clostridium *spp. count. For the most of analyzed parameters, the correlations were observed only when the values of control group (without induced hypercholesterolemia) were not considered.

**Table 4:** Correlation (r value) between bacterial populations, lipid parameters and lesion size

Bacterial population	Total Cholesterol	non-HDL-C	HDL-C	ox-LDL Ab	Lesion size
*Enterococcus *spp.	**- 0.98 **(0.19)	**-0.98 **(0.18)	**0.97 **(0.66)	**-0.90 **(0.45)	**-0.84 **(0.09)
*Lactobacillus *spp.	**- 0.99 **(0.37)	**-0.99 **(0.32)	**0.95 **(0.57)	**-0.96 **(-0.33)	**-0.78 **(0.26)
*Bifidobacterium *spp.	**- 0.99 **(0.27)	**-0.99 **(0.27)	**0.90 **(0.57)	**-0.99 **(-0.40)	**-0.81 **(0.19)
Enterobacteria	0.60 (0.27)	0.6022 (-0.27)	**-0.79 **(-0.57)	0.40 (0.07)	0.43 (-0.24)
*Clostridium *spp.	-0.49 (-0.49)	-0.49 (-0.50)	0.37 (0.41)	**-0.76 **(0.73)	**-0.76 **(-0.66)

Correlation coefficients with an r superior of 0.7 are shown in bold face type

Values including control animals are presented in parentheses.

## Discussion

Daily ingestion of aqueous soy extract fermented with *Enterococcus faecium *CRL 183 and *Lactobacillus helveticcus *416, supplemented or not with isoflavones, was found to reduce the risk of cardiovascular disease by improving the lipid profile and inhibiting oxLDL Ab formation. Earlier studies have shown the hypolipemiant and anti - atherogenic properties of this product in various animal models and clinical trials [8-11].

In the colon, the microbiota is comprised mainly of anaerobes such as *Bacteroides *spp., *Porphyromonas *spp., *Bifidobacterium *spp., *Lactobacillus *spp. and *Clostridium *spp. The composition of human microbiota remains relatively constant throughout the adult life, but it may be influenced by age, diet and socio-economic conditions and, mainly, by the use of antibiotics [24,25].

Some bacterial genera in the gut, such as *Bifidobacterium *spp. and *Lactobacillus *spp., are considered beneficial to the host, while others can be potentially pathogenic [26,27]. In this context, there is a consensus among researchers that several human disease states have benefited from the use of probiotics, most notably, diarrhea, some inflammatory bowel diseases, infectious disorders, irritable bowel syndrome and dyslipidemia [25].

After 60 days of the experiment, intake of probiotic soy product was related to significant increases (P < 0.05) in *Lactobacillus *spp. and *Bifidobacterium *spp. counts indicating that the presence of the probiotic microorganisms (*E. faecium *CRL183 and *L. helveticus *416) stimulated the growth of beneficial microorganisms (*Lactobacillus *spp. and *Bifidobacterium *spp.), since the unfermented product was not able to increase the populations of these fecal bacteria genera. Similar results were observed by other researchers, when rats on a beef-based diet were treated with the same fermented product, without isoflavone supplementation [26].

The increase in the population of intestinal bifidobacteria represents a beneficial effect for the host, since these microorganisms are involved in the production of short chain fatty acids (SCFA), reduction of intestinal pH, reduction of putrefaction products, inhibition of the growth of pathogens and immunomodulation [28].

The population of *Enterococcus *spp. decreased only in the hypercholesterolemic group (H). However, it is important to emphasize that the animals under treatment with the probiotic product (HF and HIF) exhibited the largest increases in number of microrganisms of this genus, demonstrating the probable persistence in the gut of the probiotic *E. faecium *strain during the feeding period. The health properties of *E. faecium *CRL 183, used as the starter culture in the fermented soy product, have been extensively studied and include: inhibition of breast and colon cancer [29,30], prevention of osteoporosis, modulation of the immune system [31] and hypocholesterolemic effect [7-10].

The enterobacteria group is represented mainly by *Escherichia coli, Salmonella *spp., *Shigella *spp., *Yersinia enterocolitica, Klebsiella *spp., *Proteus *spp. and *Citrobacter *spp. and is related to intestinal and extra-intestinal infections. In this study, all the animals groups showed reduction in the enterobacteria population, with the most expressive results being obtained with probiotic fermented soy product (HF) ingestion. The inhibition of potentially pathogenic bacteria by a probiotic could be related to the production of various acids, hydrogen peroxide or bacteriocins, the competition for nutrients or adhesion receptors, anti-toxin action and stimulation of the immune system [32-34].

We also observed a reduction on *Clostridium *spp. after ingestion of unfermented soy product and isoflavone-supplemented soy product. However, the fermented soy product alone (HF) did not alter the *Clostridium *spp. population. In an earlier study, it was found that the same fermented soy product led to an increase in the number of viable cells of *Clostridium *spp. in the feces of rats fed on a beef-based diet [26]. The possible explanation for this observation was that the *E. faecium *CRL 183 or the metabolites generated during fermentation promote the increase in the *Clostridium *spp. population. *Clostridium *spp. is a major component of normal anaerobic microbiota and some species may cause infectious diseases in humans [35]. However, in this study the identification of bacteria to species level was not done and it is thus not possible to decide whether the rise in the *Clostridium *spp. population is prejudicial or not.

Cardiovascular diseases are related to dyslipidemia (high levels of total cholesterol and low-density lipoprotein and low levels of high-density lipopprotein). The present study showed a strong correlation between microbiota composition and lipid profile. Population of *Enterococcus *spp., *Lactobacillus *spp. and *Bifidobacterium *spp. were negatively associated with total cholesterol, non-HDL-cholesterol, autoantibodies against oxidized LDL (oxLDL Ab) and lesion size. *Clostridium *spp. viable cells in the feces were negatively associated with antibodies against oxidized LDL (oxLDL Ab) and lesion size. HDL-C levels were positively associated with *Lactobacillus *spp., *Bifidobacterium *spp. and *Enterococcus *spp. populations.

Several studies have reported the beneficial effects of bifidobacteria and lactobacilli on lipid metabolism, when they are administered as probiotics or when their growth is stimulated by prebiotics [4,36-38]. Martinez et al (2009) [4] showed that consumption of grain sorghum lipid extract (GSL) improved the high-density lipoprotein equilibrium (HDL/non-HDL cholesterol) and this effect was associated with alteration in gut microbiota. *Bifidobacterium *spp. population exhibited a strong positive correlation with plasma cholesterol levels (r = 0.75; P = 0.001). Wang et al. (2009) [39] showed that ingestion of a diet supplemented with lyophilized *L. plantarum *MA2 (10^11 ^CFU/day) significantly lowered serum total cholesterol, low-density lipoprotein cholesterol, and triglycerides level in mice. The authors also observed an increase in the *Lactobacillus *spp. and *Bifidobacterium *spp. populations.

*In vitro *experiments, have demonstrated that *E. faecium *CRL183 reduces cholesterol by 53.85% [40] and 51.20% [41]. We have also reported that the soy product fermented with *E. faecium *CRL 183 and *Lactobacillus helveticus *416 can improve the lipid parameters in animal and human tests [7-9].

The mechanism of hypolipemiant action, although not completely known, involves assimilation of cholesterol, deconjugation of bile salts and fermentation of non-digestible carbohydrates from the diet, producing short-chain fatty acids [42,43].

The first hypothesis suggests that some microorganisms may assimilate cholesterol or incorporate this substance into the cell membrane, but this is not the consensus [44]. A second hypothesis considers that some bacterial species (such as bifidobacteria, lactobacilli, clostridia and streptococci) exhibit bile salt hydrolase activity being able to hydrolyze bile acids. Cholic and deoxycholic acids, bile acids produced from cholesterol by hepatocytes, are conjugated with glycine and taurine, respectively [45]. Desconjugated bile acids are not absorbed by the large intestine and are excreted through the feces and urine. In this situation, more cholesterol is used to synthesize bile acids, leading to a fall in blood cholesterol levels [37,45,46]. Finally, some bacterial species in the large intestine ferment unabsorbed carbohydrates to produce short-chain fatty acids (SCFA).

Acetate is the major SCFA produced, but the amount of propionate and butyrate varies. Several studies show that acetate increases total cholesterol while propionate reduces the hypercholesterolemic effect caused by acetate [45,47]. Butyrate is known to inhibit liver cholesterol synthesis and provide a source of energy for human colon epithelial cells, while propionate is involved in the inhibition of the synthesis of fatty acids in the liver, thereby lowering the rates of triacylglycerol secretion and the rate of cholesterol synthesis, which could lead to the improvement of plasma cholesterol levels [48]. The hypolipidemic effect of probiotic bacteria probably involves production of sufficient propionate and butyrate to offset the effects of acetate. An increase in the number of lactic acid bacteria, such as lactobacilli and bifidobacteria in the gut was correlated with a higher concentration of lactic acid, that may be converted in acetic and butyric acid [49]. In an *in vitro *experiment, Meimandipou et al, (2009) [50] found that *L. agilis *JCM 1048 and *L. salivarius *ssp. salicinius JCM 1230 increased the total anaerobes, lactobacilli and bifidobacteria population after 24 h incubation and enhanced the production of lactate, propionate and butyrate. In the present work, SCFA were not analyzed and further studies are necessary to investigate this point. However, we believe that a beneficial modification of the microbiota composition, observed on consumption of the fermented soy product, with or without isoflavones, could collaborate to increase of the propionate and butyrate production, so that cholesterol concentrations may be altered positively.

Oxidative modification of LDL induces the formation of immunogenic epitopes in the LDL, leading to the generation of antibodies against oxidized LDL (oxLDL Ab) [51]. We found that generation of autoantibodies against ox LDL was highest (P < 0.05) in the hypercholesterolemic group (H) and that the intake of soy fermented products (HF and HIF) prevented the rise in this parameter during the experiment. The reduction in non-HDL-C concentrations (LDL+VLDL+IDL), with a consequent reduction in LDL particles available for oxidation, observed in the HF and HIF groups could, partially, explain this effect. The importance of oxLDL Ab in atherogenesis remains unclear.

Infections have been thought to cause or promote atherosclerosis by increasing pro-atherosclerotic changes in vascular cells. These changes involve a rise in scavenger receptor expression, enhancement in uptake of cholesterol and modified low-density lipoprotein and an increase in the expression of adhesion molecules and inflammatory cytokines, leading to atherosclerotic plaque vulnerability [52]. Considering that atherosclerosis is a chronic immune inflammatory disease, it is possible that increased populations of *Bifidobacterium *spp., *Lactobacillus *spp. and *Enterococcus *spp., microorganisms that exhibit immunoregulatory properties, may be linked to the development of the atherosclerotic lesion [29,53].

## Conclusions

The results of this study suggest that daily ingestion of the probiotic soy product, supplemented or not with isoflavones, may contribute to a beneficial balance of the fecal microbiota. The findings also indicate that the modulation of microbiota is associated with an improved cholesterol profile and inhibition of atherosclerotic plaque formation.

## Abbreviations

CVD: Cardiovascular Disease;CHD: Coronary Heart Disease; C: control, H: hypercholesterolemic, HUF: hypercholesterolemic plus unfermented soy product, HF: hypercholesterolemic plus fermented soy product; HIF: hypercholesterolemic plus isoflavone-supplemented fermented soy product (HIF);TC: Total Cholesterol; HDL-C: High Density Lipoprotein Cholesterol; LDL-C: Low Density lipoprotein Cholesterol; VLDL: Very Low Density Lipoprotein Cholesterol; n-DHL-C: Non High Density Lipoprotein Cholesterol; oxLDL: oxidized Low Density Lipoprotein; oxLDL Ab: Autoantibodies against oxidized Low Density Lipoprotein, SCFA: short-chain fatty acids.

## Competing interests

The authors declare that they have no competing interests.

## Authors' contributions

DCUC: was involved in design, data collection, drafting the manuscript and revising it critically for important intellectual content.

JYS, DSPA, RCV, NDPS, MNR, RAP: participated in data collection, interpretation of results and drafting the manuscript.

EAR: was involved in design, drafting the manuscript and revising it critically for important intellectual content.

All authors read and approved the final manuscript.
